# Surgical management of giant pituitary adenomas: institutional experience and clinical outcomes of 94 patients

**DOI:** 10.3389/fonc.2023.1255768

**Published:** 2023-11-20

**Authors:** Daibo Ke, Ling Xu, Danyang Wu, Shaocheng Yang, Shun Liu, Mingxiang Xie, Shunwu Xiao

**Affiliations:** ^1^ Department of Neurosurgery, The Affiliated Hospital of Zunyi Medical University, Zunyi, China; ^2^ Graduate School, Zunyi Medical University, Zunyi, China

**Keywords:** giant pituitary adenomas, surgical management, endoscopic transsphenoidal, transcranial, combined approach

## Abstract

**Background:**

Giant (with a diameter of at least 40 mm and a volume of at least 10 cm^3^) pituitary adenomas (GPAs) are intricate tumors that pose considerable difficulty for surgical removal. While endoscopic transsphenoidal surgery (ETS) is a commonly employed technique for these destructive tumors, its effectiveness may be restricted in cases where invasion into multiple compartments is present, leading to limited resection.

**Methods:**

A retrospective review was conducted on the clinical records of 94 patients diagnosed with GPAs who had undergone surgical resection from 2014 to 2022. An analysis was conducted on the outcomes of the surgical and clinical procedures.

**Results:**

In this group, the average size of the tumor before surgery was 44.6 ± 5.6 mm (range, 40–73 mm), and the volume was 25. 5± 16.6 cm^3^ (range, 10–20.67 cm^3^). Of the total number of patients, 72 (76.6%) underwent a single ETS, 12 (12.8%) opted for transcranial surgery (TCS), and 10 (10.6%) chose a combined method. Gross total resection (GTR) was successfully performed in 49 (68.1%), 3 (25.0%), and 8 (80.0%) patients who underwent each surgical approach. Seventy-four (78.7%) patients had improved vision, 20 (21.3%) were unchanged, and none had deterioration. Twenty-two patients (23.4%) experienced a total of 43 complications, which comprised hormonal insufficiency (11/94, 11.7%), diabetic insipidus (6/88, 6.8%), electrolyte disorders (7/94, 7.4%), cerebrospinal fluid leakage (5/94, 5.3%), meningitis (8/94, 8.5%), and hydrocephalus (6/94, 6.4%). The GTR, subtotal resection (STR), and partial resection (PTR) rates were 63.8% (60/94), 21.3% (20/94), and 14.9% (14/94), respectively. Throughout the follow-up duration, 18.1% (17/94) of patients required reoperation and/or adjuvant radiation treatment as a result of tumor regrowth or inadequate biochemical remission of functioning GPAs.

**Conclusion:**

ETS remains the optimal surgical option for most GPAs and generally offers safe and efficient tumor resection. However, a combined approach with TCS remains a requirement in cases that are not suitable for treatment with a single ETS. To achieve optimal tumor removal and minimize the occurrence of surgical complications, a flexible combination of ETS and TCS is recommended based on the characteristics of the tumor.

## 1 Introduction

Mass effect symptoms and inappropriate hormonal secretion are the main manifestations of giant pituitary adenomas (GPAs), which make up approximately 5%–15% of all pituitary adenomas (1–3). Surgery for GPAs presents a significant challenge and is accompanied by considerable perioperative complications due to their substantial size and diverse growth patterns (4–6).

Endoscopic transsphenoidal surgery (ETS) is widely acknowledged as the most effective therapy and has significantly improved the success rate of gross total resection (GTR) during surgery for pituitary adenoma (7–9); however, complete removal of adenomas occupying multiple compartments is unlikely (4, 6, 10). While transcranial surgery (TCS) is not commonly used as the main procedure for GPAs, it provides enhanced accessibility to adenomas that exhibit extensive extrasellar invasion, are situated in the frontal region, or surround the arteries of the circle of Willis (11, 12). However, TCS allows limited intrasellar tumor resection, especially when the sella turcica is nearly normal-sized (13–15). Therefore, combining ETS and TCS in a single operation has recently been advocated to overcome these limitations (16–18).

This article discusses our surgical approach to treating 94 GPAs. We characterize the lesions and discuss their applications and limitations of respective treatment strategies.

## 2 Materials and methods

A review was conducted on the clinical data of patients who had undergone surgery for GPAs at the Affiliated Hospital of Zunyi Medical University between January 2014 and December 2022. GPAs were classified as growth with a maximum size of ≥4 cm and a volume of ≥10 cm^3^ on MRI before surgery. Approval for this study was granted by the Ethics Committee at the Affiliated Hospital of Zunyi Medical University.

### 2.1 Patient characteristics

Out of a total of 94 individuals, 50/94 (53.2%) were women and 44/94 (46.8%) were men. The average age was 52.6 ± 10.8 years (ranging from 26 to 73 years old). Regarding the type of tumor, nonfunctioning pituitary adenomas were present in 83/94 (88.3%) patients, while growth hormone (GH)-secreting adenomas were found in 6/94 (6.4%) individuals. Additionally, 3/94 (3.2%) patients had prolactin (PRL)-secreting adenomas, and 2/94 (2.1%) patients had thyroid-stimulating hormone (TSH)-secreting adenomas. The preoperative complaints included visual impairment (81/94, 86.2%), headache (36/94, 38.3%), abducens nerve palsy (8/94, 8.5%), acromegaly (6/94, 6.4%), diabetes insipidus (6/94 6.4%), and oculomotor nerve palsy (4/94, 4.3%). A preoperative endocrine disorder in ≥1 axis was found in 57/94 cases, including adrenal insufficiency in 29/94 (30.8%), hypothyroidism in 21/94 (22.3%), hypogonadism in 19/94 (20.2%), hyperprolactinemia in 18/94 (19.1%), elevated GH in 10/94 (10.6%), panhypopituitarism in 8/94 (8.5%), and elevated TSH in 2/94 (2.1%) (**Table 1**).

**Table 1:** Clinical characteristics of the 94 patients with giant pituitary adenomas.

Clinical feature	Number		%
Total number	94		100
Sex			
Male	50		53.2
Female	44		46.8
Age			
Mean ± SD (years)		52.6 ± 10.8	
Tumor type			
Nonfunctioning adenoma	83		88.3
GH-secreting adenoma	6		6.4
PRL-secreting adenoma	3		3.2
TSH-secreting adenoma	2		2.1
Preoperative symptoms			
Visual impairment	81		86.2
Headache	36		38.3
Abducent nerve palsy	8		8.5
Acromegaly	6		6.4
Diabetes insipidus	6		6.4
Oculomotor nerve palsy	4		4.3
Preoperative pituitarism disorder			
Hypoadrenalism	29		30.8
Hypothyroidism	21		22.3
Hypogonadism	19		20.2
Hyperprolactinemia	18		19.1
Elevated GH	10		10.6
Panhypopituitarism	8		8.5
Elevated TSH	2		2.1
Prior treatment			
ETS	3		3.2
TCS	2		2.1
Radiotherapy	3		3.2

GH, growth hormone; PRL, prolactin; TSH, thyroid-stimulating hormone; ETS, endoscopic transsphenoidal surgery; TCS, transcranial surgery.

Eight (8.5%) individuals had received previous medical interventions, specifically a transcranial procedure, a transsphenoidal procedure, or radiotherapy (with two, three, and three patients, respectively). The average length of follow-up was 39.8 ± 23.4 months, ranging from 7 to 96 months.

### 2.2 Radiologic evaluation

All patients underwent preoperative MRI, which showed an average maximum size of 42 ± 6 mm and an average volume of 28.9 ± 12.2 cm^3^, calculated using the formula (length × width × height)/2 (19). Tumors were categorized based on their extension beyond the suprasellar and parasellar regions using the Hardy grading system (20). This classification comprised 46/94 (48.9%) grade III tumors, 34/94 (36.2%) grade IV tumors, and 14/94 (14.9%) grade V tumors. Tumor invasion of the cavernous sinus was determined based on the Knosp grades (21), which consisted of 18/94 (19.1%) grade I tumors, 37/94 (39.4%) grade II tumors, 29/94 (30.9%) grade III tumors, and 10/94 (10.6%) grade IV tumors (**Table 2**).

**Table 2:** Radiological characteristics of the 94 giant pituitary adenomas.

Radiological characteristics	Number		%
Total number	94		100
Maximum diameter			
Mean ± SD (mm)		44.6 ± 5.6	
Volume (cm^3^)		25.5 ± 16.6	
Hardy grade			
Grade C	46		48.9
Grade D	34		36.2
Grade E	14		14.9
Knosp grade			
Grade I	18		19.1
Grade II	37		39.4
Grade III	29		30.9
Grade IV	10		10.6

The extent of resection (EOR) was categorized as gross total resection (GTR; no detectable remaining tumor), subtotal resection (STR; > 80%), or partial resection (PTR; ≤ 80%) (5).

### 2.3 Surgical approach

**Figure 1:**
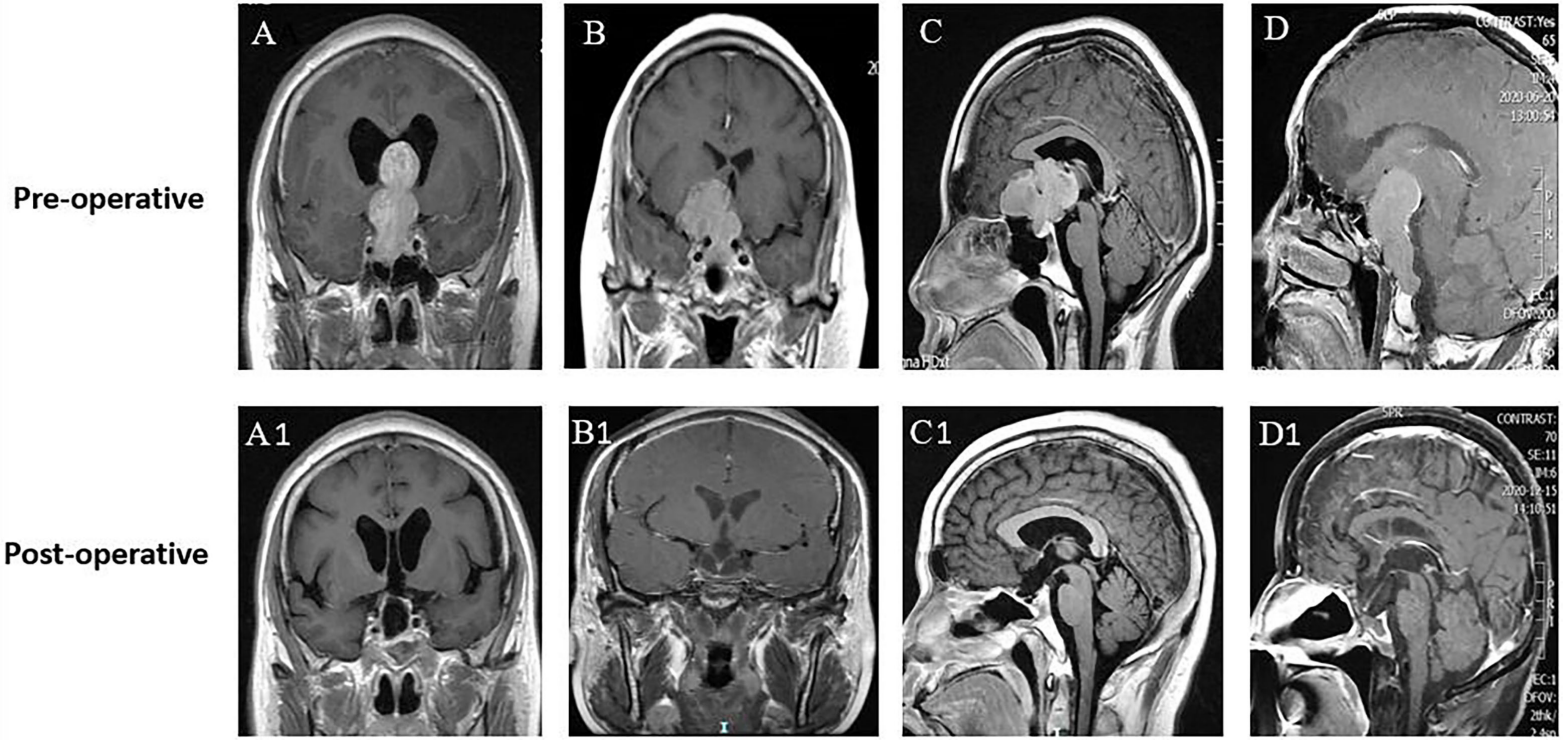
Different directions and extents of suprasellar extension did not influence the EOR. **(A–D)** Preoperative MR images of 4 patients with GPAs. All patients were characterized by no sign of cavernous sinus invasion (Knosp grade 0-I). **(A)** Gourd-like GPA with extreme suprasellar extension into the third ventricle and obstructive hydrocephalus. **(B)** Dumbbell-shaped GPA with lateral extension and displacement of the left anterior cerebral artery. **(C)** Lobulated GPA with forward extension into the planum sphenoidale. **(D)** Oval-shaped GPA with suprasellar and posterior fossa extension by dorsum sellae and clivus erosion. **(A1–D1)** Postoperative MR images of A–D, respectively, showing no visible residual tumor (GTR) in these GPAs of different suprasellar extensions.

**Figure 2:**
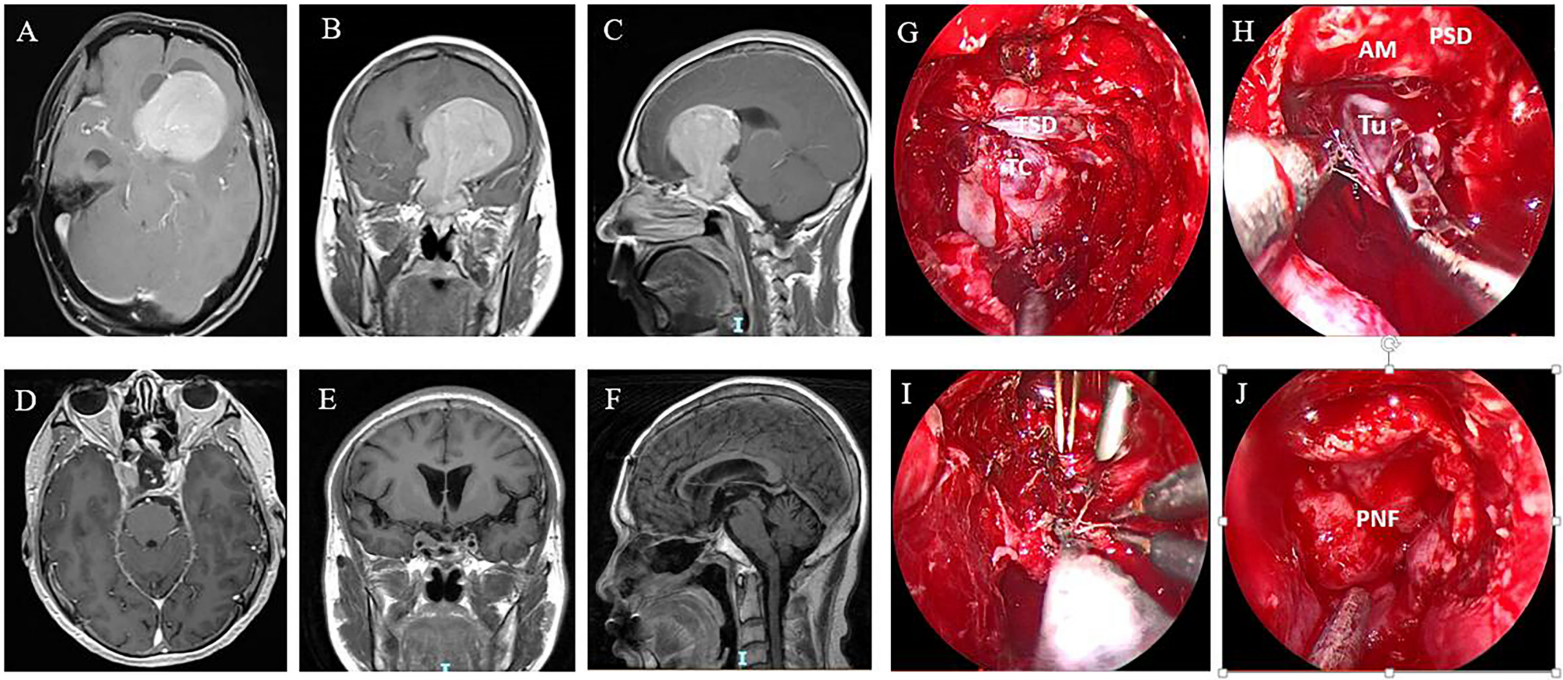
A GPA in a 46-year-old male patient. Preoperative axial **(A)**, coronal **(B)**, and sagittal **(C)** MR images show the tumor extending into the left lateral ventricle. The patient underwent ETS and a combined left frontal craniotomy via a transcortical approach. Postoperative MR images showing that the tumor was gross totally resected **(D–F)**. **(G–J)** Intraoperative images of the surgical stages. **(G)** Endoscopic view after sellar opening. The dura was quite thin and adhered closely to the tumor capsule. **(H)** The suprasellar part of the tumor did not descend after intrasellar tumor resection and needed to be mechanically delivered from the cranial path. **(I)** The tumor was removed sufficiently with ETS and simultaneous TCS. **(J)** The skull base defect was reconstructed with an autologous fat graft, a pedicled nasoseptal flap, and oxidized cellulose with fibrin glue. TSD, tuberculum sella dura; TC, tumor capsule; Tu, tumor; AM, arachnoid membrane; PSD, planum sphenoidale dura; PNF, pedicled nasoseptal flap.

Patients in this research underwent removal of a pituitary tumor using precise imaging assistance and utilization of microvascular Doppler probes if needed to locate the carotid arteries and determine the extent of bone removal. The approach was chosen individually for each case to achieve complete tumor resection, maintain normal hormone secretion, preserve pituitary function, and eliminate the potential for recurrence. Initially, an attempt was made to achieve adequate tumor resection for the majority of GPAs using a single approach involving a regular or expanded ETS (**Figure 1**). A complementary TCS was adopted if the suprasellar part could not be satisfactorily and safely resected due to its irregular shape, para-midline extension or optic apparatus, and/or cerebral artery encasement (**Figure 2**). Residual tumors within the cavernous sinus were routinely treated with radiation therapy to avoid serious complications. Only in cases where a tumor displayed significant invasion beyond the sella turcica and had a small portion within it was a single TCS employed.

### 2.4 Skull base reconstruction

Skull base reconstruction is a crucial procedure. The central component of reconstruction is multilayered closure, which is designed according to the defect size, and the volume and location of the CSF leak. Small defects with low-flow CSF leaks can be closed with a Gelfoam inlay and a second inlay of artificial meninges tucked under the border of the dura. As a third layer, the pedicled vascularized nasoseptal flap can be positioned as an onlay and further supported by gelatin sponges and silver ion gauze. In cases with larger defects, it is preferred to use an inlay composed of both fat and fascia lata, an onlay of bone flap (*in situ* bone flap, nasal septum, and vomer), and a third layer composed of the same material as in cases with small defects. A rigid buttress is indispensable for tumors with extended dural resection. In large defects with high-flow CSF leaks, an additional onlay graft may be applied to cover the dura margin as another layer of reinforcement.

### 2.5 Follow-up

We evaluated the postoperative outcomes of all patients who presented with clinical manifestations and who underwent visual tests, endocrine examinations, and MRIs. Routine examination was performed 1 day, 7 days, 3 months, and 6 months postoperatively and annually thereafter. Visual tests, including visual acuity and visual field tests, were conducted. Pituitary function was assessed with a comprehensive endocrinological examination. Specific criteria were adopted for defining biochemical remission of GH secreting tumors (IGF-I in the age-adjusted normal range, and a random GH level <1 mcg/L or nadir GH levels <0.4 mcg/L) (22) and PRL secreting tumors (baseline PRL levels <200 mcg/L in female patients and 150 mcg/L in male patients) (23). The extent of tumor resection was calculated on the basis of intraoperative findings and postoperative MRI.

### 2.6 Statistical analysis

Data statistics and analysis were conducted using SPSS software version 17.0. Means and standard deviations or medians and ranges were used to present continuous variables, whereas numbers and percentages were used to present categorical variables. The analysis of categorical variables involved the utilization of either the chi-square test or Fisher’s exact test. Student’s *t*-test was used to analyze continuous variables. A statistically significant result was determined if the *p*-value was less than 0.05.

## 3 Results

### 3.1 Surgical outcomes

Seventy-two (76.6%) patients underwent a single ETS, including 10 (10.6%) who underwent a single standard transsphenoidal approach and 62 (66.0%) who underwent an extended transsphenoidal procedure. Twelve (12.8%) patients underwent a single TCS and 10 (10.6%) underwent multiple surgical procedures, including 8 (8.5%) who underwent simultaneous combined ETS and TCS and 2 (2.1%) who underwent a two-stage surgery, both of which were performed with an ETS at first and a subsequent TCS 3 months later (**Table 3**).

**Table 3:** Statistical analysis of different variables on extent of resection.

Variable	Total		GTR		STR		PTR		*p*
	No.	%	No.	%	No.	%	No.	%	
Age (years)									
Mean ± SD			51.4 ± 5.8		46 ± 6.7		52 ± 8.2		0.674
Sex									0.810
Female	50	50/94 (53.2%)	31	31/50 (62.0%)	14	14/50 (28.0%)	5	5/50 (10.0%)	
Male	44	44/94 (46.8%)	29	29/44 (65.9%)	6	6/44 (13.6%)	9	9/44 (20.5%)	
Maximum diameter (mm)									
Mean ± SD	44.6 ± 5.6		44.10 ± 5.33		46.30 ± 6.01		44.64 ± 6.22		0.191
Tumor volume									
Mean ± SD	25.5 ± 16.6		22.70 ± 15.45		32.73 ± 18.19		26.95 ± 16.00		0.096
Tumor type									0.793
Nonfunctioning adenoma	83	83/94 (88.3%)	51	51/83 (61.4%)	19	19/83 (35.8%)	13	13/83 (15.7%)	
GH-secreting adenoma	6	6/94 (6.4%)	4	4/6 (66.7%)	1	1/6 (16.7%)	1	1/6 (16.7%)	
PRL-secreting adenoma	3	3/94 (3.2%)	3	3/3 (100%)	0	–	0	–	
TSH-secreting adenoma	2	2/94 (2.1%)	2	2/2 (100%)	0	–	0	–	
Hardy stages									0.560
C	46	46/94 (48.9%)	28	28/46 (60.9%)	10	10/46 (21.7)	8	8/46 (17.4)	
D	34	34/94 (36.2)	25	25/34 (73.5%)	6	6/34 (17.6%)	3	3/34 (8.8%)	
E	14	14/94 (14.9%)	7	7/14 (50.0%)	4	4/14 (28.6%)	3	3/14 (21.4%)	
Knosp grades									0.085
I	18	18/94 (19.1%)	13	13/18 (72.2%)	3	3/18 (16.7%)	2	2/18 (11.1%)	
II	37	37/94 (39.4%)	28	28/37 (75.6%)	5	5/37 (13.5%)	4	4/37 (10.8%)	
III	29	29/94 (30.9%)	16	16/29 (55.2%)	9	9/29 (31.0%)	4	4/29 (13.8%)	
IV	10	10/94 (10.6%)	3	3/10 (30.0%)	3	3/10 (30.0%)	4	4/10 (40.0%)	
Knosp grades group									0.037
I, II	55	55/94 (58.5%)	41	41/55 (74.5%)	8	8/55 (14.5%)	6	6/55 (10.9%)	
III, IV	39	39/94 (41.5%)	19	19/39 (48.7%)	12	12/39 (30.8%)	8	8/39 (20.5%)	
Previous surgery									0.955
Yes	8	8/94 (8.5%)	5	5/8 (62.5%)	2	2/8 (25%)	1	1/8 (12.5%)	
No	86	86/94(91.5%)	55	55/86 (69.4%)	18	18/86(20.9%)	13	13/86 (15.1%)	
Surgical approach									0.004
ETS	72	72/94 (76.6%)	49	49/72 (68.1%)	15	15/72 (20.1%)	8	8/72 (11.1%)	
TCS	12	12/94 (12.8%)	3	3/12 (25.0%)	3	3/12 (25.0%)	6	6/12 (50.0%)	
ETS+TCS	10	10/94 (10.6)	8	8/10 (80%)	2	2/10 (20%)	0	–	

GH, growth hormone; PRL, prolactin; TSH, thyroid-stimulating hormone; ETS, endoscopic transsphenoidal surgery; TCS, transcranial surgery.

p-values <0.05 were considered statistically significant.

The symbol “-” means no significant figures can be used when under analysing, because the numerator and/or denominator was “0” when a percentage formula was adopted.

**Figure 3:**
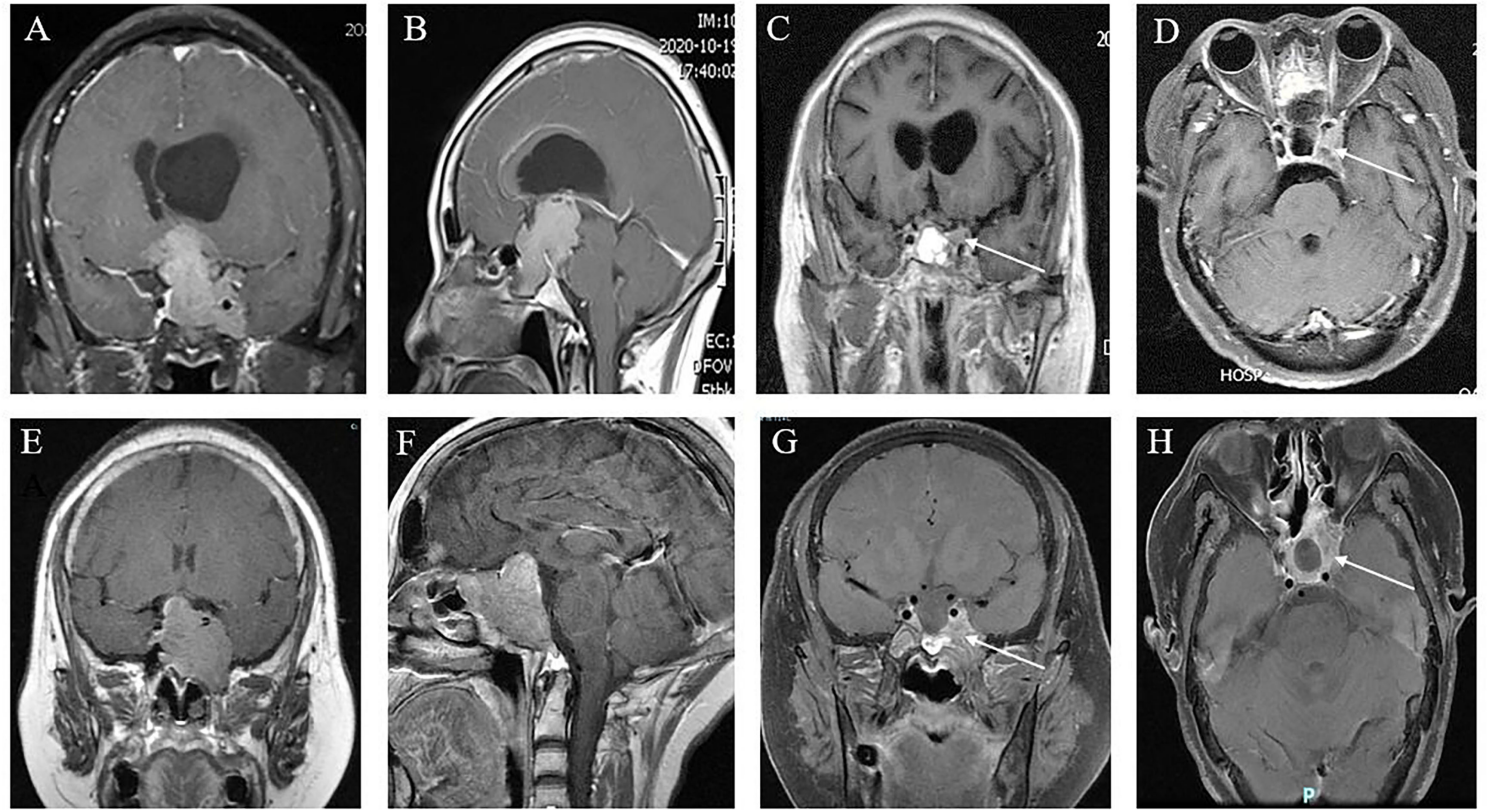
Cavernous sinus invasion limits the GTR of GPA. **(A, B)** Preoperative coronal **(A)** and sagittal views **(B)** showing a nonfunctional GPA with significant invasion of the sphenoid sinus and left cavernous sinus, encasement of the left internal carotid artery, and compression of the third ventricle with obstructive hydrocephalus; **(C, D)** 6 months postoperatively, approximately 5% of tumors remained in the cavernous sinus (white arrow), which was subtotal resection. **(E, F)** Coronal **(E)** and sagittal **(F)** MR images of a patient with a giant GH adenoma. This tumor is also sphenoid sinus occupying, cavernous sinus invasion, and ICA surrounding. **(G, H)** Three months after ETS, demonstrating that approximately 25% of tumors remained (white arrow), which were partially resected.

The first postoperative MRI showed GTR, STR, and PTR in 63.8% (60/94), 21.3% (20/94), and 14.9% (14/94) of patients, respectively. The maximum diameter and tumor volume were not different among the PTR, STR, and GTR groups (*p* > 0.05). The EOR was not affected by extension into the suprasellar region, as patients with different degrees and directions of suprasellar extension (Hardy stages III, IV, and V) achieved similar resection rates (*p* > 0.05) (**Figures 1**, **2**). The EOR in tumors classified as Knosp grade III–IV was notably lower than that in tumors classified as Knosp grade I–II (*p* = 0.037), which indicated that the outcome of surgery was significantly influenced by cavernous sinus invasion (**Figure 3**). The rate of PTR was significantly lower in ETS and combined surgery groups (*p* = 0.004).The EOR was not significantly predicted by factors such as sex, age, endocrine function, or previous surgical history (**Table 3**).

### 3.2 Clinical outcome and complications

Among 81 patients with preoperative visual impairment, 71 (83.1%) experienced visual improvement, and the remaining 10 (12.3%) cases were unchanged. Out of the 12 individuals who had cranial nerve palsy before surgery, 10 (83.3%) experienced marked improvement and 2 (16.7%) maintained a similar condition; no surgical visual impairment and other cranial nerve injury occurred in this series.

There were 79 patients with available hormonal information at the last follow-up control. Of 29 patients with preoperative hypoadrenalism, 11/29 (37.9%) returned to normal after surgery. Among 21 patients with hypothyroidism, 9/21 (42.9%) showed gross recovery. Eight out of 19 (42.1%) patients with preoperative gonadal dysfunction experienced a notable postoperative improvement. In those cases of panhypopituitarism, a partial improvement was found in 4/8 (50%). Hormone replacement therapy was administered as an alternative for patients with constant pituitary hormonal deficits. Early endocrinologic remission was not achieved in any of six somatotroph adenomas and three prolactinomas. Except for one somatotroph adenoma and one prolactinoma without total tumor resection that failed to achieve biochemical remission and was under adjuvant medical treatment and/or radiotherapy, all the somatotroph adenomas and prolactinomas with GTR achieved biochemical remission after an average of 26 months of adjuvant medical treatment. Surgical resection of two TSH-secreting adenomas led to GTR and early biochemical remission without further need for medical therapy.

In total, 43 complications were experienced by 22 patients, accounting for 23.4%. Out of the 11 patients, postoperative hypopituitarism emerged as the prevailing condition. Within a span of 3 months, eight of these people experienced recovery through the implementation of hormone replacement therapy. Antibiotic treatment and lumbar drainage were administered to a total of eight individuals diagnosed with meningitis. Electrolyte disorders occurred in seven patients, and six were accompanied by diabetic insipidus. All these disorders disappeared before discharge. Six patients with preoperative hydrocephalus were not relieved due to postoperative hemorrhage of the residual tumor; three of these patients had transient external ventricular drainage, and the remaining patients selected conservative treatment with close observation. Within a span of 2 weeks, lumbar drainage effectively treated all five patients who experienced new-onset cerebrospinal fluid (CSF) leakage. No deaths or mortal complications occurred in this case series (**Table 4**).

**Table 4:** Clinical outcomes and complications according to the type of surgical approach.

Clinical outcomes	All cases		ETS (*n* = 72)		TCS (*n* = 12)		ETS+TCS (*n* = 10)		*p*
	No.	%	No.	%	No.	%	No.	%	
Visual outcome									
Improved	71	71/81 (83.1%)	58	58/65 (87.7%)	8	8/10 (80.0%)	5	5/6 (83.3%)	0.379
Unchanged	10	10/81 (12.3%)	7	7/65 (10.8%)	2	2/10 (20.0%)	1	1/6 (16.7%)	0.651
Remission rates									
Hyperprolactinemia	18	18/18 (100%)	9	9/10 (90.0%)	5	5/5 (100%)	3	3/3 (100%)	0.127
Elevated GH	10	10/10 (100%)	9	9/10 (90.0%)	0	–	0	–	–
Elevated TSH	2	2/2 (100%)	2	2/2 (100%)	0	–	0	–	–
Cranial nerve palsy									
Improved	10	10/12 (83.3%)	7	7/8 (87.5%)	2	2/3 (66.7%)	1	1/1 (100%)	0.098
Unchanged	2	2/12 (16.7%)	1	1/8 (12.5%)	1	1/3 (33.3%)	0	–	0.513
Complications									
Number of patients	22	22/94 (23.4%)	12	12/72 (16.7%)	5	5/12 (41.7%)	5	5/10 (50%)	0.091
Panhypopituitarism	5	5/49 (10.2%)	3	3/33 (9.1%)	1	1/10 (10.0%)	1	1/6 (16.7%)	0.625
Hypoadrenalism	4	4/67 (6.0%)	3	3/51 (5.9%)	0	–	1	1/7 (14.3%)	0.184
Hypogonadism	1	1/54 (1.9%)	1	1/39 (2.6%)	0	–	0	–	–
Hypothyroidism	1	1/73 (1.4%)	1	1/54 (1.9%)	0	–	0	–	–
Meningitis	8	8/94 (8.5%)	4	4/72 (5.6%)	2	2/12 (16.7%)	2	2/10 (20.0%)	0.171
Electrolyte disorders	7	7/94 (7.4%)	3	3/72 (4.2%)	1	1/12 (8.3%)	2	2/10 (20.0%)	0.087
Diabetic insipidus	6	6/88 (6.8%)	4	4/67 (6.0%)	1	1/12 (8.3%)	1	1/9 (11.1%)	0.828
Hydrocephalus	6	6/94 (6.4%)	5	5/72 (6.9%)	1	1/12 (8.3%)	0	–	0.626
CSF leakage	5	5/94 (5.3%)	4	4/72 (5.6%)	0	–	1	1/10 (10.0%)	0.572
Tumor regrowth									
STR group	6	6/20 (30%)	5	5/15 (33.3%)	1	1/3 (33.3%)	0	–	0.287
PTR group	9	9/14 (64.3%)	4	4/8 (50.0%)	5	5/6(83.3%)	0	–	0.002

GH, growth hormone; TSH, thyroid-stimulating hormone; ETS, endoscopic transsphenoidal surgery; TCS, transcranial surgery; CSF, cerebrospinal fluid; STR, subtotal resection; PTR, partial resection.

p-values <0.05 were considered statistically significant.

The symbol “-” means no significant figures can be used when under analysing, because the numerator and/or denominator was “0” when a percentage formula was adopted.

Following a median follow-up period of 39.8 ± 23.4 months (ranging from 7 to 96 months), MRI showed no tumor recurrence in the GTR patients. Of the 20/94 (21.3%) cases showing STR, tumor regrowth was found in 6/20 (30%) patients. However, up to 64.3% (9/14) of patients in the PTR group experienced residual tumor regrowth (**Table 4**). All 15 recurrent tumors, in addition to 2 functional GPAs that had not achieved remission with residual tumor, were first treated with radiotherapy. At the last follow-up, four (23.5%) patients encountered tumor regrowth and subsequently underwent reoperation.

## 4 Discussion

Nonfunctional macroadenoma adenomas make up over 70% of all pituitary adenomas (PAs), while GPAs contribute to approximately 10% of cases (6, 16, 24). They are generally symptomatic as a result of adjacent structure compression, resulting in visual impairment, ocular movement dysfunction, hypopituitarism, and hydrocephalus (10, 25). Except prolactinomas, which can be treated with dopamine agonists, surgical management is the preferred choice for the majority of GPAs (26, 27). Maximizing the safe removal of the tumor is the main objective of surgery, although achieving complete resection can be difficult and potentially dangerous in cases where the tumor extends significantly beyond the sella and invades the cavernous sinus (28, 29).

Because transsphenoidal surgery is more accessible and allows better exposure than the transcranial route when dealing with cavernous sinus and infradiaphragmal adenoma components, it has become the mainstay of GPA management (6, 9, 27). We prefer the endoscopic to the microsurgical transsphenoidal approach in the surgical treatment of GPAs due to a wider surgical corridor and high-quality visualization (28–30). Furthermore, angled endoscopes enable deeper exploration into the sellar fossa and facilitate resection of adenoma components that are otherwise inaccessible when using the microsurgical transsphenoidal approach (14, 31, 32). A regular ETS is rarely used only when the tumor grows with midline extension and intact diaphragma sellae (6, 9). If the diaphragma sellae is breached and the tumor is multilobulated or “snowman” shaped, an extended ETS may be ideal to remove the portions with mild anterior, posterior, or lateral extension (31, 33). In our series, 72/94 (76.6%) patients were treated by a single ETS, and we achieved a high GTR rate of 68.1% and a low complication rate of 16.7%. All these outcomes were on par with those of other authors and were proven to be much better for effectively managing these GAPs than TCS (31, 34, 35). However, single ETS for radical resection of the supra or parasellar compartment of GPAs with extreme paramidline, intraventricular, and skull base extension is difficult, which explained why 23/72 (32%) GPAs in the single-ETS group were incompletely resected. Furthermore, a recent study of single ETS on 64 GPAs reported that up to 43/64 (67%) patients had intracranial remnants, and 17/43 (32%) of these tumors underwent TCS or delayed second ETS (5).

While not commonly used as the main surgical procedure for GPAs, TCS continues to be a favorable choice for relieving pressure on the optic pathways and conveniently reaching the tumor components through either extreme paramedian or frontal extension (11, 12, 36). We chose primary TCS for patients with normal-sized sella turcica and breached diaphragma sellae with predominantly suprasellar extension and multilobulated appearance (6, 17), which indicated that major tumor resection contraindicated the transsphenoidal corridor. Any approach, including transcortical, transcallosal, frontotemporal, and orbitozygomatic approaches, that enables resection along the long axis of the tumor may be preferred (6, 36). In our series, 12/94 (12.8%) patients were treated with a single TCS. Except for the limitation of intrasellar tumor resection, most intracranial compartments with different Hardy grades could be totally resected via any of the transcranial approach. However, in line with prior research, the current series shows that TCS is linked to a reduced rate of GTR (25.0%) and an increased rate of postoperative complications (41.7%) than ETS and combined surgery because of persistent optic nerve traction and difficulty in manipulating the supplying vasculature underlying the tumor (14, 25, 37).

If there is GPA that presents with both infra- to intrasellar and suprasellar extension, especially with an anterior communication artery complex, optic chiasma encasement, and lateral extension beyond the circle of Willis, causing severe cranial hypertension, there should be access above and below the tumor to allow resection (34, 38, 39). Combined ETS and TCS can provide unrestricted dissection and direct protection of neurovascular structures through the transcranial space, and adequate hemostasis can be achieved by blocking the supplying vasculature at the early stage from the ETS corridor (16–18). Our research found that the rate of GTR using the combined approach was 80%, which surpassed the rates achieved by any of the individual methods. The postoperative complication rate (20%) was similar to that in the single ETS group. Therefore, combined surgery is likely to be a preferred strategy for these complex GPAs (16, 39). Some authors have suggested two-stage surgery for these patients. They generally performed ETS surgery first, followed by TCS 3 months later (6, 17, 29). However, in contrast to the simultaneously combined ETS and TCS approach, two-stage surgery poses a high risk (5%–12.9% in the literature) of postoperative hemorrhage and subsequent mass effect and/or neurological deterioration because of insufficient transsphenoidal tumor resection (14, 25, 34). Therefore, we are more likely to adopt a simultaneously combined ETS and TCS approach for GPAs that could not be gross totally resected through a single approach.

## 5 Conclusions

Managing GPAs surgically continues to be a difficult task. For most GPAs, utilizing an expanded ETS can be a secure and effective choice as the primary option. If the adenoma components are initially inaccessible through ETS, simultaneous TCS can be utilized to accomplish complete or near-total removal of the tumor while minimizing the chances of complications and recurrence. Long-term control of inoperable tumor remnants necessitated the use of adjuvant radiation and/or medical treatment. Therefore, GPAs often require a multimodality treatment strategy with a flexible combination of individually tailored approaches.

## Data availability statement

The original contributions presented in the study are included in the article/supplementary materials. Further inquiries can be directed to the corresponding author.

## Author contributions

DK: Writing – original draft, Writing – review & editing. LX: Data curation, Formal analysis, Investigation, Methodology, Writing – original draft. DW: Data curation, Investigation, Writing – original draft, Formal analysis. SY: Data curation, Writing – original draft, Formal analysis. SL: Data curation, Writing – original draft, Formal analysis. MX: Conceptualization, Writing – original draft. SX: Conceptualization, Supervision, Writing – original draft.

## References

[1] Goel A Nadkarni T Muzumdar D Desai K Phalke U Sharma P. Giant pituitary tumors: a study based on surgical treatment of 118 cases. Surg Neurol (2004) 61(5):436–45; discussion 445-6. doi: 10.1016/j.surneu.2003.08.036 15120215

[2] Mortini P Barzaghi R Losa M Boari N Giovanelli M. Surgical treatment of giant pituitary adenomas: strategies and results in a series of 95 consecutive patients. Neurosurgery (2007) 60(6):993–1002; discussion 1003-4. doi: 10.1227/01.neu.0000255459.14764.ba 17538372

[3] de Paiva Neto MA Vandergrift A Fatemi N Gorgulho AA Desalles AA Cohan P. Endonasal transsphenoidal surgery and multimodality treatment for giant pituitary adenomas. Clin Endocrinol (Oxf) (2010) 72(4):512–9. doi: 10.1111/j.1365-2265.2009.03665.x 19555365

[4] Di Ieva A Rotondo F Syro LV Cusimano MD Kovacs K. Aggressive pituitary adenomas–diagnosis and emerging treatments. Nat Rev Endocrinol (2014) 10(7):423–35. doi: 10.1038/nrendo.2014.64 24821329

[5] Micko A Agam MS Brunswick A Strickland BA Rutkowski MJ Carmichael JD. Treatment strategies for giant pituitary adenomas in the era of endoscopic transsphenoidal surgery: a multicenter series. J Neurosurg (2022) 136(3):776–85. doi: 10.3171/2021.1.jns203982 34388714

[6] Makarenko S Alzahrani I Karsy M Deopujari C Couldwell WT. Outcomes and surgical nuances in management of giant pituitary adenomas: a review of 108 cases in the endoscopic era. J Neurosurg (2022), 1–12. doi: 10.3171/2021.10.jns21659 35061979

[7] Sanai N Quiñones-Hinojosa A Narvid J Kunwar S. Safety and efficacy of the direct endonasal transsphenoidal approach for challenging sellar tumors. J Neurooncol (2008) 87(3):317–25. doi: 10.1007/s11060-007-9512-2 18094936

[8] Nakao N Itakura T. Surgical outcome of the endoscopic endonasal approach for non-functioning giant pituitary adenoma. J Clin Neurosci (2011) 18(1):71–5. doi: 10.1016/j.jocn.2010.04.049 20851609

[9] Koutourousiou M Gardner PA Fernandez-Miranda JC Paluzzi A Wang EW Snyderman CH. Endoscopic endonasal surgery for giant pituitary adenomas: advantages and limitations. J Neurosurg (2013) 118(3):621–31. doi: 10.3171/2012.11.jns121190 23289816

[10] Gondim JA Almeida JP Albuquerque LA Gomes EF Schops M. Giant pituitary adenomas: surgical outcomes of 50 cases operated on by the endonasal endoscopic approach. World Neurosurg (2014) 82(1-2):e281–90. doi: 10.1016/j.wneu.2013.08.028 23994073

[11] Dolenc VV. Transcranial epidural approach to pituitary tumors extending beyond the sella. Neurosurgery (1997) 41(3):542–50; discussion 551-2. doi: 10.1097/00006123-199709000-00007 9310970

[12] Youssef AS Agazzi S van Loveren HR. Transcranial surgery for pituitary adenomas. Neurosurgery (2005) 57(1 Suppl):168–75; discussion 168-75. doi: 10.1227/01.neu.0000163602.05663.86 15987585

[13] Mohr G Hardy J Comtois R Beauregard H. Surgical management of giant pituitary adenomas. Can J Neurol Sci (1990) 17(1):62–6. doi: 10.1017/s0317167100030055 2311019

[14] Komotar RJ Starke RM Raper DM Anand VK Schwartz TH. Endoscopic endonasal compared with microscopic transsphenoidal and open transcranial resection of giant pituitary adenomas. Pituitary (2012) 15(2):150–9. doi: 10.1007/s11102-011-0359-3 22038033

[15] Couldwell WT. Transsphenoidal and transcranial surgery for pituitary adenomas. J Neurooncol (2004) 69(1-3):237–56. doi: 10.1023/b:neon.0000041886.61149.ab 15527094

[16] Leung GK Law HY Hung KN Fan YW Lui WM. Combined simultaneous transcranial and transsphenoidal resection of large-to-giant pituitary adenomas. Acta Neurochir (Wien) (2011) 153(7):1401–8; discussion 1408. doi: 10.1007/s00701-011-1029-y 21533660 PMC3111555

[17] Han S Gao W Jing Z Wang Y Wu A. How to deal with giant pituitary adenomas: transsphenoidal or transcranial, simultaneous or two-staged? J Neurooncol (2017) 132(2):313–21. doi: 10.1007/s11060-017-2371-6 28074324

[18] Kuga D Toda M Ozawa H Ogawa K Yoshida K. Endoscopic endonasal approach combined with a simultaneous transcranial approach for giant pituitary tumors. World Neurosurg (2019) 121:173–9. doi: 10.1016/j.wneu.2018.10.047 30336293

[19] Monga SP Wadleigh R Sharma A Adib H Strader D Singh G. Intratumoral therapy of cisplatin/epinephrine injectable gel for palliation in patients with obstructive esophageal cancer. Am J Clin Oncol (2000) 23(4):386–92. doi: 10.1097/00000421-200008000-00016 10955870

[20] Hardy J Vezina JL. Transsphenoidal neurosurgery of intracranial neoplasm. Adv Neurol (1976) 15:261–73. 945663

[21] Knosp E Steiner E Kitz K Matula C. Pituitary adenomas with invasion of the cavernous sinus space: a magnetic resonance imaging classification compared with surgical findings. Neurosurgery (1993) 33(4):610–7; discussion 617-8. doi: 10.1227/00006123-199310000-00008 8232800

[22] Giustina A Chanson P Bronstein MD Klibanski A Lamberts S Casanueva FF. A consensus on criteria for cure of acromegaly. J Clin Endocrinol Metab (2010) 95(7):3141–8. doi: 10.1210/jc.2009-2670 20410227

[23] Melmed S Casanueva FF Hoffman AR Kleinberg DL Montori VM Schlechte JA. Diagnosis and treatment of hyperprolactinemia: an Endocrine Society clinical practice guideline. J Clin Endocrinol Metab (2011) 96(2):273–88. doi: 10.1210/jc.2010-1692 21296991

[24] Mete O Cintosun A Pressman I Asa SL. Epidemiology and biomarker profile of pituitary adenohypophysial tumors. Mod Pathol (2018) 31(6):900–9. doi: 10.1038/s41379-018-0016-8 29434339

[25] Sinha S Sharma BS. Giant pituitary adenomas–an enigma revisited. Microsurgical treatment strategies and outcome in a series of 250 patients. Br J Neurosurg (2010) 24(1):31–9. doi: 10.3109/02688690903370305 20158350

[26] Liu JK Couldwell WT. Contemporary management of prolactinomas. Neurosurg Focus (2004) 16(4):E2. doi: 10.3171/foc.2004.16.4.3 15191331

[27] Agrawal A Cincu R Goel A. Current concepts and controversies in the management of non-functioning giant pituitary macroadenomas. Clin Neurol Neurosurg (2007) 109(8):645–50. doi: 10.1016/j.clineuro.2007.06.007 17686573

[28] Han S Ding X Tie X Liu Y Xia J Yan A. Endoscopic endonasal trans-sphenoidal approach for pituitary adenomas: is one nostril enough? Acta Neurochir (Wien) (2013) 155(9):1601–9. doi: 10.1007/s00701-013-1788-8 23736939

[29] Guinto-Nishimura GY Caballero-Delgado S Eguiluz-Meléndez AG Ortega-Porcayo LA Valencia-Ramos C Aragon-Arreola JF. Combined endoscopic transsphenoidal and tubular retractor-assisted transventricular approach for giant pituitary adenomas. World Neurosurg (2021) 155:e761–9. doi: 10.1016/j.wneu.2021.08.135 34500097

[30] Guvenc G Kizmazoglu C Pinar E Imre A Kaya I Bezircioglu H. Outcomes and complications of endoscopic versus microscopic transsphenoidal surgery in pituitary adenoma. J Craniofac Surg (2016) 27(4):1015–20. doi: 10.1097/scs.0000000000002684 27213744

[31] Fallah N Taghvaei M Sadaghiani S Sadrhosseini SM Esfahanian F Zeinalizadeh M. Surgical outcome of endoscopic endonasal surgery of large and giant pituitary adenomas: an institutional experience from the Middle East. World Neurosurg (2019) 132:e802–11. doi: 10.1016/j.wneu.2019.08.004 31404693

[32] Kuo CH Yen YS Wu JC Chang PY Chang HK Tu TH. Primary endoscopic transnasal transsphenoidal surgery for giant pituitary adenoma. World Neurosurg (2016) 91:121–8. doi: 10.1016/j.wneu.2016.03.092 27060516

[33] Jamaluddin MA Patel BK George T Gohil JA Biradar HP Kandregula S. Endoscopic endonasal approach for giant pituitary adenoma occupying the entire third ventricle: surgical results and a review of the literature. World Neurosurg (2021) 154:e254–63. doi: 10.1016/j.wneu.2021.07.022 34293521

[34] Zada G Du R Laws ER Jr. : Defining the "edge of the envelope": patient selection in treating complex sellar-based neoplasms via transsphenoidal versus open craniotomy. J Neurosurg (2011) 114(2):286–300. doi: 10.3171/2010.8.jns10520 20815698

[35] Marigil Sanchez M Karekezi C Almeida JP Kalyvas A Castro V Velasquez C. Management of giant pituitary adenomas: role and outcome of the endoscopic endonasal surgical approach. Neurosurg Clin N Am (2019) 30(4):433–44. doi: 10.1016/j.nec.2019.05.004 31471050

[36] Guo F Song L Bai J Zhao P Sun H Liu X. Successful treatment for giant pituitary adenomas through diverse transcranial approaches in a series of 15 consecutive patients. Clin Neurol Neurosurg (2012) 114(7):885–90. doi: 10.1016/j.clineuro.2012.01.033 22326130

[37] Musleh W Sonabend AM Lesniak MS. Role of craniotomy in the management of pituitary adenomas and sellar/parasellar tumors. Expert Rev Anticancer Ther (2006) 6 Suppl 9:S79–83. doi: 10.1586/14737140.6.9s.S79 17004861

[38] Juraschka K Khan OH Godoy BL Monsalves E Kilian A Krischek B. Endoscopic endonasal transsphenoidal approach to large and giant pituitary adenomas: institutional experience and predictors of extent of resection. J Neurosurg (2014) 121(1):75–83. doi: 10.3171/2014.3.jns131679 24785323

[39] D'Ambrosio AL Syed ON Grobelny BT Freda PU Wardlaw S Bruce JN. Simultaneous above and below approach to giant pituitary adenomas: surgical strategies and long-term follow-up. Pituitary (2009) 12(3):217–25. doi: 10.1007/s11102-009-0171-5 PMC3321841 19242807

